# Application of Calcium Citrate in the Manufacture of Acid Rennet Cheese Produced from High-Heat-Treated Goat’s Milk from Spring and Autumn Season

**DOI:** 10.3390/molecules27175523

**Published:** 2022-08-27

**Authors:** Małgorzata Pawlos, Agata Znamirowska-Piotrowska, Magdalena Kowalczyk, Grzegorz Zaguła

**Affiliations:** 1 Department of Dairy Technology, Institute of Food Technology and Nutrition, College of Natural Sciences, University of Rzeszow, Cwiklinskiej 2D, 35-601 Rzeszow, Poland; 2 Department of Bioenergetics, Food Analysis and Microbiology, Institute of Food Technology and Nutrition, College of Natural Sciences, University of Rzeszow, Zelwerowicza 4, 35-601 Rzeszow, Poland

**Keywords:** goat’s milk, pasteurization, fermentation, cheese, acid rennet coagulation, calcium, mineral composition, texture, organoleptic evaluation

## Abstract

The stability of milk proteins is affected by changes in the pH value of milk, the heating temperature, and the addition of calcium compounds or chelating agents, which can cause alterations in calcium distribution. The purpose of this study was to determine the potential of the use of calcium citrate to manufacture fresh acid rennet cheese from high-temperature-pasteurized goat’s milk (90 °C, 15 s) from the spring and autumn season and the effect of the calcium dose used on the physicochemical and organoleptic properties of the cheese. Autumn milk was found to be a richer source of total solids, confirming the effect of the production season on milk quality. The applied doses of calcium did not cause the denaturation of goat milk proteins and allowed pasteurization to take place at 90 °C for 15 s. The addition of calcium citrate resulted in a significant increase in the pH value of milk and cheese compared to the control sample. Adding 15 and 20 mg of Ca 100 g^−1^ to milk as citrate had the most beneficial effect on increasing protein retention in cheese in both seasons, showing a rise from 1.33% to 2.40%. The production season significantly influenced the cheese yield. The control goat cheese from the autumn season showed a 6.85% higher yield compared to the spring cheese. An increase in cheese yield was also observed as the calcium dose of milk increased. The content of micro- and microelements in cheese was affected by the production season. The addition of calcium citrate to milk resulted in a significant increase in the calcium content of cheese—from 120.83 to 147.45 mg 100 g^−1^ in the spring season and from 130.66 to 151.21 mg 100 g^−1^ in the autumn season. Increasing the dose of calcium increased the hardness of cheese samples by 1.37 N in the spring and 0.90 N in the autumn. The organoleptic evaluation showed that adding calcium to milk did not significantly affect the organoleptic characteristics of goat cheese.

## 1. Introduction

Acid rennet curd formation is driven by partially treating casein micelles with rennet, which enhances acid coagulation and leads to the formation of a more solid and firm acid gel [1]. Rennet has an acidic nature with an optimum activity in a slightly acidic environment. Therefore, the action of lactic acid bacteria in this phase is crucial, as they are required to rapidly release enough lactic acid to lower the pH and create an appropriate environment for the optimal activity of rennet. The fermentation of milk by acidification leads to changes in the colloidal system of milk [2]. Calcium occurs in soluble and colloidal forms in milk, with some of the soluble fractions being ionic. The stability of milk proteins is affected by changes in the pH value of milk, the heating temperature, and the addition of calcium compounds or chelating agents, which can cause changes in calcium distribution. According to Umeda and Aoki [3] and Ozcan-Yilsay et al. [4], the addition of calcium reduces the hydration of casein micelles and increases micellar density. The addition of calcium to milk has a positive effect on the rennet coagulation process. It improves interactions during aggregation by neutralizing electrostatic repulsion between casein micelles and is involved in the formation of bridges between caseins [1].

The modification of the calcium equilibrium in milk by adding calcium chloride, which is commonly used in cheese manufacture, results in increased levels of ionic calcium, which decreases the ethanol stability of milk [5,6]. In the composition of milk compounds, citric acid salts (citrates and hydrogen citrates of calcium, magnesium, and potassium) play a crucial role. Approximately 90% of the citric acid in milk is made up of soluble compounds that are an essential component of the buffer system. The binding of calcium and magnesium in the form of citrates increases the heat stability of milk proteins [7,8]. Additionally, Boumpa et al. [9] observed a reduction in ionic calcium levels and increased ethanol stability after the sterilization and UHT treatment of goat’s milk after adding citrate to milk.

In acid curd cheese manufacture, a large portion of whey proteins is removed from whey during draining. Despite their excellent nutritional value, whey proteins are characterized by high water retention and gelling properties in their denatured phase [10]. The heat treatment of milk for denaturing whey proteins and binding them to the casein fraction is one of the effective methods with which to recover whey proteins in cheese production technology. According to Guyomarc’h et al. [10], the pasteurization of milk at temperatures above 60 °C induces the denaturation of whey proteins followed by aggregation through hydrophobic interactions and disulfide—thiol exchange. In this process, heat-induced aggregates are formed either on the surface of casein micelles or in the serum phase of milk. These aggregates mainly contain whey proteins and κ-casein. However, micelle-associated aggregates may also contain trace amounts of αs_2_-casein [10,11]. According to Kelly et al. [12], the high-heat treatment of milk prolongs the coagulation time and reduces rennet gel, leading to impaired syneresis. The adverse effect on coagulation is attributed to the inhibition of κ-casein hydrolysis by rennet due to the presence of β-lactoglobulin/κ-casein complex on the micelle surface, which reduces the availability of κ-casein for the coagulant. Milk coagulation is also affected by the reduced reactivity of micelles with attached denatured whey proteins and the reduced calcium concentration in the micelles [12]. However, the gel-forming properties of heat-treated milk can be partially restored by reducing the pH, increasing the temperature of the milk during coagulation, increasing the amount of rennet added, and adding calcium compounds to the cheese milk [12].

The chemical composition of goat’s milk fluctuates and is influenced by numerous factors, including genetic (breed), environmental (nutrition, season), and physiological (stage of lactation, udder health) aspects. The lactation stage in goats is associated with the production season and usually begins in the first months of the year in Polish goats [13,14]. According to Barłowska et al. [13], the season of production is an essential factor differentiating the content of the basic components of goat’s milk, and a higher content is found in the autumn–winter season, at the end of lactation.

Various methods have been used in goat’s milk to achieve proper curd firmness by increasing the total solids and protein content, such as mixing it with bovine milk or fortifying it with whey protein concentrate [15]. Goat’s milk gels are poorer and softer and provide a lower cheese yield [16]. Due to the fragile texture of goat’s milk curd and the difficulty of processing it, acid curd cheese is usually produced with the addition of rennet, since the addition of this enzyme increases the hardness of the curd [17]. Moreover, our previous study [18] showed that by increasing the heat treatment temperature of the goat’s milk and adding calcium in the form of various compounds before the milk pasteurization process, the physicochemical properties of goat’s milk gel could be improved.

This study aimed to determine the potential of the use of calcium citrate to manufacture fresh acid rennet cheese from high-temperature-pasteurized goat’s milk (90 °C, 15 s) from the spring and autumn season and the effect of production season and calcium dose on the physicochemical and organoleptic properties of the cheese.

## 2. Results and Discussion

### 2.1. Quality of Raw Goat’s Milk

The seasonal variability of goat’s milk composition, including the content of macro- and microelements, strongly influences goat’s milk’s technological and sensory properties [19,20]. Variability in the chemical composition of milk depends on many environmental, genetic, and physiological factors and may be related to the season and the lactation stage of the goat [17]. Barłowska et al. [13], Park et al. [21], and Zamberlin et al. [22] showed that goat’s milk is a more valuable source of macroelements, including calcium, and some microelements than cow milk. A study by Guo et al. [23] showed that the calcium content steadily decreased from approximately 0.16 to 0.14% at week 20 of lactation and then increased to 0.16% at around week 40 of lactation. Cheese milk composition is crucial in cheese production, affecting cheese yield and production efficiency. Cheese composition depends on the microbiological and chemical composition of the milk, the cheese-making technology used, the conditions, and the ripening time. Since milk fat and protein are the main components of cheese, the quality of the product is strongly influenced by their concentration in milk [24].

Goat’s milk obtained in the autumn season was characterized by a significantly higher content of total protein (by 0.57 g 100 g^−1^), fat (by 0.69 g 100 g^−1^), and total solids (by 1.34 g 100 g^−1^. Furthermore, the goat’s milk from the autumn season contained significantly higher levels of micro- and macroelements than that from the spring season (*p* ≤ 0.05) (Table 1). Goat’s milk contains an average of 3.4 g 100 g^−1^ protein, 3.8 g 100 g^−1^ fat, 4.1 g 100 g^−1^ lactose, and 0.8 g 100 g^−1^ ash [22]. In the study of Kljajevic et al. [25], in mid-lactation, the main composition parameters such as fat, protein, non-fat solid, and lactose contents were significantly lower than those in early and late lactation. Kędzierska-Matysek et al. [26] reported that the chemical composition and concentrations of most of the elements tested varied throughout lactation, which was linked to the season of production. Milk from the final stage of lactation was characterized by a higher content of protein (3.14 g 100 g^−1^), fat (3.79 g 100 g^−1^), and total solids (12.15 g 100 g^−^^1^) as well as Ca (124.13 mg 100 g^−1^), Na (46.45 mg 100 g^−1^), Mg (16.94 mg 100 g^−1^), and Mn (0.01 mg 100 g^−1^). In a study by Norris et al. [27], it was demonstrated that during the autumn season, goats produce milk with a higher concentration of protein by 0.12% and a higher concentration of fat by 0.20%.

EU regulation [28] allows a maximum of 1.5 million CFU mL^−1^ in goat’s milk. The total bacterial count (TBC) shown in Table 1 was much lower, indicating that the evaluated milk meets the criteria of Regulation 1662/2006 [28]. In the autumn season, the number of somatic cells (SCC) was higher by about 576,000 compared to that in the spring season. The decrease in the cytological quality of goat’s milk at the end of lactation was also shown by Barłowska et al. [13] and Danków et al. [29]. A similar trend was observed by Brodziak et al. [30], showing a lower cytological quality of milk in the autumn—winter season. This was probably related to the stage of lactation, because at the end of the lactation period, with a decrease in milk yield, there is an increase in the number of somatic cells.

The pH value and the freezing point of goat’s milk were in accordance with results obtained by Kljajevic et al. [25], Mayer and Fiechter [31], Park et al. [21], and Strzałkowska et al. [32].

### 2.2. Effect of Calcium Dose on the pH Value of Goat’s Milk after Pasteurization

Thermal treatment is one of the most common processing methods used in the dairy industry and may destroy microorganisms in raw milk, which can ensure food safety and extend the shelf life of products [33]. The heat treatment of milk can cause adverse effects that include changes in milk acidity and lactose degradation. Thermal processes can also affect the mineral balance of milk, especially calcium and phosphate, causing calcium and phosphate to transition to a colloidal state [34]. Furthermore, the heat treatment of milk can result in the aggregation of whey protein and casein and is one of the main chemical changes that occur during the heat treatment of goat milk [33].

**Figure 1:**
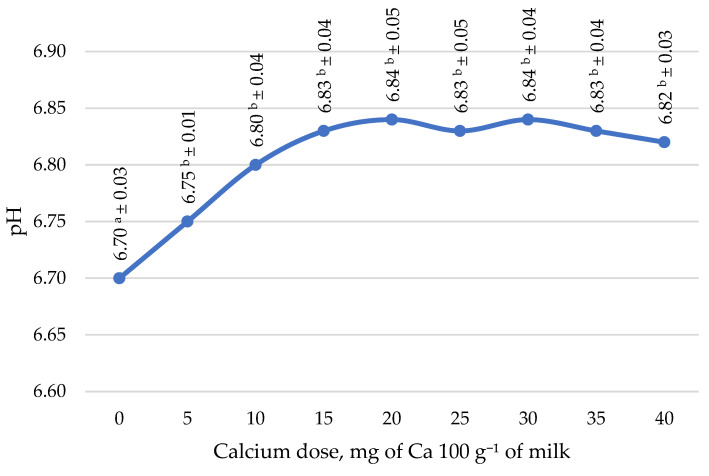
Effect of calcium dose on the pH value of goat’s milk after pasteurization (90 °C, 15 s). Mean ± standard deviation; ^a,b^ means with different letters indicate statistically significant differences at *p* < 0.05; 5 samples × 9 batches = 45.

Figure 1 shows the pH values after the pasteurization of control goat milk (0 mg Ca 100 g^−1^) and with the addition of calcium citrate at doses of 5, 10, 15, 20, 25, 30, 35, and 40 mg Ca 100 g^−1^ of milk. The application of calcium doses ranging from 0 mg to 40 mg of calcium per 100 g of milk was carried out to determine whether goat milk proteins would denature during the heat treatment of milk at 90 °C for 15 s. The applied doses of calcium did not cause the denaturation of goat milk proteins and allowed pasteurization at 90 °C for 15 s. The addition of calcium citrate resulted in a significant increase in the pH value of the milk compared to the control sample (*p* < 0.05). The addition of 5–20 mg Ca 100 g in the form of citrate increased the pH value, while further increases in calcium doses did not increase the pH value of milk. This was due to the properties of this calcium compound, as reported by Ziarno et al. [35]. According to these authors [35], an essential advantage of poorly water-soluble compounds (calcium citrate) is neutrality to milk proteins, even at increased temperature, so they can be added to milk before pasteurization without concern for decreasing the heat stability of proteins and precipitation.

The thermal stability of milk is affected by both the type of calcium compound used and the type of milk [36]. According to Smiths and Brouwershaven [37], Ca^2+^ may contribute to the heat-induced binding of low concentrations of β-lactoglobulin to casein micelles by forming complex Ca^2+^ bonds with ionic groups in proteins, thus reducing intermolecular repulsion and promoting the formation of intermolecular hydrophobic bonds. Furthermore, the calcium content in milk is also significantly correlated with the gelling properties of milk proteins [2]. Calcium added to milk can react with proteins, causing sedimentation and gelation, especially if the milk is heat-treated.

### 2.3. Quality of Acid Rennet Goat Cheese with Calcium Addition

#### 2.3.1. Physicochemical Properties and Yield of Acid Rennet Goat Cheese

The increase in the pH value of cheese with the increase in calcium dose was caused by citrate addition. This was confirmed by the correlation coefficients between the calcium dose and the pH value, which assume positive values in both seasons (Table 2). The pH of cheese samples made from milk from the autumn season was found to be significantly higher (*p* ≤ 0.05). In a study by Dmytrów et al. [38], similar pH values of goat curd were determined, i.e., 4.67 on day of production. However, after three days of storage, the pH value decreased to 4.54. Janštovà et al. [39] showed a wider range of pH from 4.75 to 5.12 in fresh goat cheese. According to many authors [38,40], differences in the acidity of dairy products are due to the activity and growth rate of the starter bacteria, as well as to the type of milk used and its chemical composition. A high proportion of proteins and minerals promotes the activity of lactic acid bacteria. Data presented by Masle and Morgan [41] also showed that the composition of goat milk depends on the lactation stage; therefore, the susceptibility of goat milk to acidification varies with the lactation stage.

A higher fat content in the milk from the autumn season also resulted in a higher fat content in the cheese, but the results were not statistically significant (*p* ≤ 0.05). However, the addition of increasing doses of calcium to milk from both seasons allowed more fat to be retained in the cheese, and this trend was confirmed by the positive correlation coefficients found (Table 2). A study by Coulon et al. [42] also indicated that rennet goat cheese made from milk obtained in early lactation showed a lower fat content in dry matter, while a higher fat level characterized cheese made in the final stage of lactation. The fresh goat cheese analyzed by Janštova et al. [39] showed a fat content ranging from 0.08% to 30.83%. In contrast, Miloradovic et al. [43] obtained goat cheese with a fat content of 17.50% to 18.50%. Acid curd cheese made from goat’s milk produced by Dmytrów et al. [38] contained 9.0% fat on the first day of storage.

The total solids content was highly dependent on protein retention and the amount of fat in the cheese. Cheese made in the autumn season from goat’s milk without calcium and with calcium citrate at a level of 20 mg Ca 100 g^−1^ was found to have a significantly higher total solids content than cheese obtained in the spring season (*p* ≥ 0.05) (Table 2). There was a tendency for the total solids content to increase with an increase in calcium dose in cheeses with citrate in both seasons, for which the calculated correlation coefficients showed positive values. The reason for the higher total solids content was probably the binding of whey proteins to casein. The study results obtained by Janštova et al. [39] showed a higher level of cheese total solids, from 44.08% to 50.05%. However, in the study of Dmytrów et al. [39], acid curd cheese from goat’s milk was characterized by a total solids content of only 27.5% on the first day of storage.

**Figure 2:**
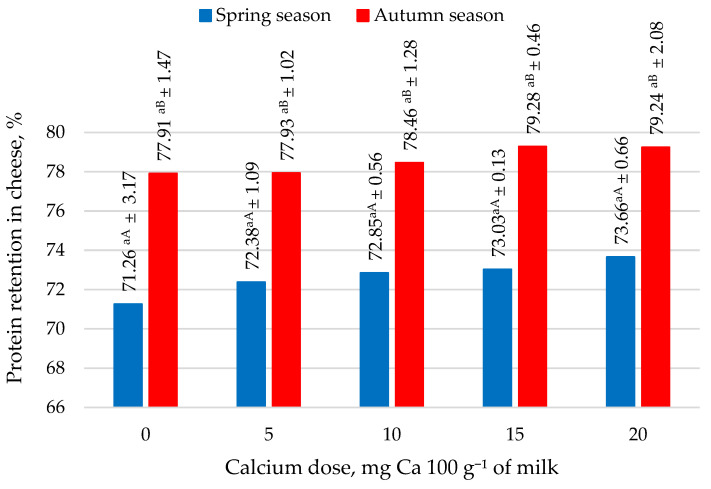
Protein retention in cheese in the spring and autumn season. Mean ± standard deviation; ^a^ means with the same lowercase letters indicate not significant differences at *p* < 0.05 depending on calcium dose in spring or autumn season; ^A,B^ means with different capital letters indicate significant differences at *p* < 0.05 depending on the season.

**Figure 3:**
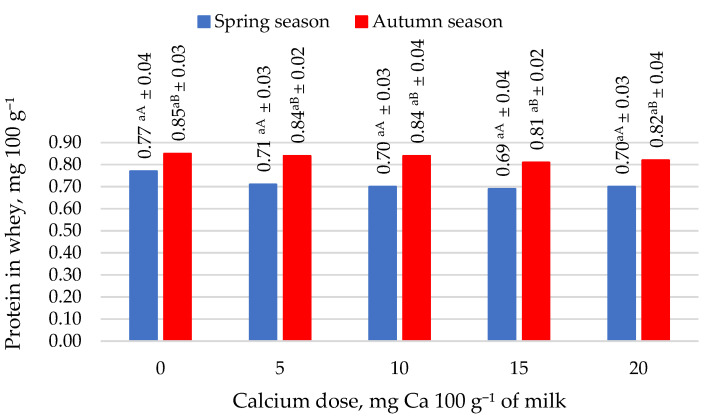
Protein concentration in whey in the spring and autumn season. Mean ± standard deviation; ^a^ means with the same lowercase letters indicate not significant differences at *p* < 0.05 depending on calcium dose in spring or autumn season; ^A,B^ means with different capital letters indicate significant differences at *p* < 0.05 depending on the season.

The protein retention of protein from milk to cheese was found to be lower in the spring season than in the autumn season (*p* ≤ 0.05) (Figure 2). This distribution was due to milk’s higher total protein content in the autumn season. The increasing addition of calcium citrate positively influenced the increasing level of protein retention in cheese. This observation implies that as the calcium dose increased, the protein content of the cheese increased. Adding 15 and 20 mg of Ca 100 g^−1^ to milk as citrate had the most beneficial effect on increasing protein retention in cheese in both seasons. A higher protein retention in cheese was correlated with lower protein migration into the whey. The analysis of the protein content of whey from the production of goat cheese from milk with added calcium confirmed this trend (Figure 3). Increasing doses of calcium in the form of citrate in a goat milk resulted in the lower protein content in whey from cheese production in both seasons. The addition of 20 mg of calcium in the form of citrate resulted in a reduction in the protein content of whey from cheese production in the spring season of 10.39% and a reduction of 4.71% in the autumn season.

The retention of valuable milk components in the final product is a process that is particularly important in acid cheese production, where a significant proportion of milk components are transferred to whey [44]. According to Koutina et al. [45], the intricate processes of protein interaction with the participation of some macroelements that occur during the calcium–thermal–acid coagulation of milk proteins could affect the degree of retention of milk components in acid curd cheese and determine their chemical composition and nutritional value. The integration of whey proteins into acid cheese is related to an increase in the nutritional value of protein caused by increasing the number of essential amino acids [46]. Siemianowski and Szpendowski [47] determined that in the traditional method of acid curd cheese production, the level of protein utilization is 75%. Using the thermal–calcium method increases total protein utilization to 90% [44]. Additionally, Bohdziewicz [48] pointed in the direction of optimizing the efficiency of whey protein utilization using the thermal–calcium method, which provides the possibility of separating up to 96% of milk proteins in the form of coprecipitates.

In acid curd cheese production, the aim is to maximize the use of milk components while increasing the production yield [49]. Yield is the number of liters of milk used to produce 1 kg of cheese [48]. Yield is one of the most important variables of economic factors in cheese production due to the different chemical composition of milk and the use of different cheese production technologies [50]. The level of protein and fat migration into the whey, as well as its degree of dispersion, depends on several factors, the most important of which include the thermal treatment of the milk, the acidity of the milk and whey, mechanical treatment, and the reheating of the cheese curd [51].

The production season significantly influenced the cheese yield. The yield of the control goat cheeses from the spring season was 15.23%, while that produced in the autumn season was 6.85% higher (Table 2). The increase in yield was also observed as the calcium dose of milk increased by 2.37% in the spring season and 1.52% in the autumn season in cheese samples made from goat’s milk with the addition of 20 mg Ca 100 g^−1^. The correlation coefficients confirmed this effect of calcium dose on cheese yield, which showed positive values in both analyzed seasons (Table 2).

The findings of many authors [52,53] indicate that cheese yield depends on the protein content of milk, particularly the casein fraction proteins. Low protein levels in processed milk reduce cheese yield and increase the cost of cheese production. In this study, the goat milk used for cheese production contained between 2.69 g 100 g^−1^ and 3.26 g 100 g^−1^ total protein (Table 1). A higher level of casein, which accounts for 2.14 g 100 g^−1^ to 3.85 g 100 g^−1^ in goat milk [17,21,23], might increase the yield of goat cheese produced during the autumn season. It should be noted that both the protein retention from milk to cheese and the protein content of whey from cheese production with the addition of calcium were also higher during the autumn season. A study by Dmytrów et al. [38] showed that the acid curd cheese from goat’s milk had a weaker firmness, which could lead to its spreading and a decreased cheese yield. Based on the experimental results, it could be concluded that as calcium dose increased, the yield and retention of protein in cheese increased, while the concentration of protein in whey decreased (Figure 2). In a study by Dmytrów et al. [38], goat acid curd cheese had the lowest yield compared to cow cheese and cheeses made from a mixture of cow and goat milk. Milodranovic et al. [43] obtained a 9.5% yield of acid rennet cheese made from goat’s milk using milk pasteurization at 60 °C for 30 min. In contrast, the cheese yield from milk heated at 80 °C for 5 min and at 90 °C for 5 min was 3.3% and 4.3% higher, respectively, than when 60 °C was used. The increase in yield of goat cheese was due not only to the formation of covalently bound protein aggregates but also the presence of both hydrophobic and electrostatic interactions between proteins.

The calcium-thermal method can be used in the production of acid and acid rennet cheese, and its application, according to Siemianowski and Szpendowski [54], makes it possible to increase the product yield by up to 10–15%. In our study, the increase in yield was only 2.18% compared to the control cheese. Fekadu et al. [55] stated that the actual yield of rennet goat cheese was higher when produced from milk from the early (May) and late (September and October) stages of lactation compared to the middle period of lactation (July and August), which is attributed to differences in the chemical composition of milk (mainly fat and protein). These authors suggest that goats are fed fresh pasture feed during the summer months, which contains more water and less solids, contributing to higher milk yields but with a lower total solids content.

#### 2.3.2. Mineral Composition of Acid Rennet Goat Cheese

According to Britten and Giroux [56], the addition of calcium ions to milk before pasteurization increases the surface area of casein micelles and enhances polymerization and aggregation of whey proteins, consequently increasing the interaction effect between casein and whey protein aggregates. When milk is heated, the transformation of soluble calcium into a colloidal form occurs, which participates in forming the casein complex with whey proteins. It is claimed that ionic bonds between phosphoric acid residues, mediated by calcium ions, determine the complexes stability of the formed between milk proteins [56].

In the experiment, adding calcium citrate increased the calcium content of goat cheese (Table 3). It was also found that the calcium content of cheese increased with an increasing calcium dose, as confirmed by significant positive correlation coefficients (*r* > 0.8). The production season in which milk was obtained significantly influenced the calcium content of the cheese. The study showed that the control cheese samples contained significantly more calcium in the autumn season than in the spring season. The addition of calcium citrate to milk at a level of 20 mg Ca 100 g^−1^ resulted in a 20.55–26.62% increase in the calcium content of cheese. According to Baran et al. [57], acid goat cheese contained 128.30 mg 100 g^−1^ of calcium, and this concentration was within the range determined in our study.

As the calcium dose increased, the potassium content of the cheese samples was found to decrease. The potassium content in cheese was slightly affected by the production season, as autumn milk showed more potassium than in the spring season. Baran et al. [57] determined a higher potassium content in acidic goat cheese (128.10 mg 100 g^−1^).

Some authors consider that magnesium in the form of colloidal phosphate can also participate in the formation of the micellar structure of casein and acid curd in acid cheese [58]. In our study, in most cases, increasing the calcium dose resulted in a lower magnesium content in cheese, as shown by negative correlation coefficients (Table 3). The magnesium content in cheese was significantly affected by production season, as cheese from milk from the autumn season was characterized by a higher magnesium content (*p* < 0.05). A lower magnesium content (11.90 mg 100 g^−1^) in sour goat cheese was shown by Baran et al. [57].

Presumably, as the ionic calcium content in milk increases, the quantity of native milk phosphorus increases, which will be integrated into the structure of the acid curd in the form of colloidal calcium phosphate [45]. The analysis of the phosphorus content of the goat cheese samples showed that as the amount of calcium added increases, the phosphorus content of the cheese increases (Table 3). Significant positive correlation coefficients were shown between the dose of introduced calcium and the phosphorus content of the cheese samples. The higher phosphorus content in autumn milk (Table 1) also resulted in a higher phosphorus content in cheese from the autumn season. The beneficial effect of using the calcium–thermal–acid method in acid cheese technology is to increase its nutritional value by increasing the amount of calcium and phosphorus in the curds.

A high concentration of trace elements such as manganese, molybdenum, and selenium was found in goat cheese samples. Their content was significantly influenced by the milk production season, as higher concentrations were determined in autumn cheese samples. Baran et al. [57] determined a lower manganese content in acid goat cheese (5.33 µg 100 g^−1^) than in our study. However, Park et al. [14] found a higher concentration of manganese in fresh goat cheese at 9.55 µg 100 g^−1^.

The high bioavailability of calcium from dairy products is mainly due to its good calcium and phosphorus ratio (Ca:P > 1) and the presence of numerous components that increase bioavailability (vitamin D, phosphopeptides released during casein hydrolysis, L-lysine, L-arginine, lactose) and the absence of components that hinder absorption [58]. In our study, the addition of calcium resulted in a calcium/phosphorus ratio greater than 1, which may contribute to the better bioavailability of macroelements.

#### 2.3.3. Texture Profile of Acid Rennet Goat Cheese

Texture is a sensory, structural, mechanical, and functional characteristic of food that can be perceived and described by the human senses, such as the senses of sight, hearing, touch, and kinesthesia [59]. The multidimensionality of texture is due to its molecular, microscopic, and macroscopic structure. The mechanical instruments used to determine food texture measure specific physical parameters (e.g., hardness, springiness, gumminess, chewiness, or elasticity). The results of instrumental measurements of food texture also relate to the sensory evaluation of products, as they are essential components that influence consumer perception and preference. Combining instrumental measurements and organoleptic analysis provides an opportunity to more thoroughly understand the sensory properties of food products [59]. Different types of cheese vary in texture, which, together with organoleptic characteristics, determines their properties and consumer acceptance [60]. The texture profile analysis (TPA) test involves exposing a sample to double compression using a probe with a larger diameter than the cheese being tested. This type of measurement simulates the operation of chewing food by human jaws, i.e., the compression that occurs in the mouth [61].

The hardness is the force required to achieve the desired deformation of the sample. The result of cheese hardness measurement is influenced by factors such as cheese compactness, water distribution in curd, homogeneity of samples, and technological processes carried out during its production [62,63]. The hardness of goat cheese with the addition of calcium in the spring season was higher than cheese from milk obtained in the autumn. Only the control cheese from the autumn season was characterized by higher hardness than cheese without calcium addition from milk from the spring season, but these differences were not statistically significant (*p* ≤ 0.05). The trend was observed that increasing calcium dosage increased the hardness of cheeses in both seasons (Table 4). This result is confirmed by the calculated positive, simple correlation coefficients between hardness and calcium dose, which indicate that the addition of increasingly higher doses of calcium increased the hardness of the cheese.

The acid goat cheese made by Dmytrów et al. [37] was soft and characterized by a hardness ranging from 0.30 N (on the production day) to 0.45 N (on the 21st day of storage). The low hardness of acid goat cheeses can be attributed to the lower αs_1_-casein content compared to cow’s milk. This fraction of casein plays an essential role during gel formation and its lower content impairs texture properties [37]. The observations of Dzwolak et al. [64] indicated that starter cultures direct fermentation processes in cheese production and affect the rate of curd syneresis. In our study, CSK mesophilic cultures (Danisco) were used, fermenting the milk for 16 to 18 h, resulting in a medium-compact curd. According to Mulawka et al. [63], an increase in acidity resulting from the metabolic activity of starter cultures results in a shrinkage of the acid gel, which leads to the occurrence of local stresses and the release of whey leading to an increase in the firmness of the curd. According to Dzwolak et al. [64], syneresis influenced the final texture of curd, as the correct ratio between the amount of casein-bound (colloidal) calcium and water-soluble calcium determined its basic structure. Therefore, increasing the amount of casein-bound calcium by adding it to the milk before heat treatment could increase the hardness of the cheese. Dmytrów et al. [65] and Mazur et al. [62] showed that an increase in protein and water content in acid cheese increases the hardness of cheese. This observation was also confirmed by the results in our study, as a 2.40% increase in protein retention in cheese made from milk with 20 mg Ca added from the spring season and 1.33% from the autumn season compared to the control samples resulted in increases in hardness of 1.29 N and 0.90 N, respectively. Cheese with the addition of calcium citrate was characterized by a higher total solids content than the control. Furthermore, a tendency was observed for the total solids to increase with the increase in the dosage of calcium added to the milk (Table 3).

Cohesiveness is characterized by the compactness of a sample, which is the strength of its internal bonds that constitute the sample’s structure [66]. The instrumental definition describes the cohesiveness as the ratio of work done to compress the sample during the first and second cycles. Cohesiveness was shown to increase with increasing calcium dosage (Table 4). This was confirmed by the calculated correlation coefficients, where positive values between calcium dosage and cohesiveness for cheese with citrate addition in both analyzed seasons were calculated. Mazur et al. [62] determined a similar level of cohesiveness, at 0.32, in acid semi-skimmed cow’s cheese, with the highest water content (74.9%). The cohesiveness of fat cottage cheese, with the lowest water content (68.7%), was 0.03 units lower than half-fat acid curd cheese. Chen et al. [67] demonstrated in their study the correlation between the number of somatic cells in milk and the cohesiveness of semi-soft goat ripening cheese. Cheese on the first day after production, made from milk with high somatic cell counts, had the highest cohesiveness (0.89) compared to cheese with medium and low somatic cell counts (0.37). The number of somatic cells in the autumn milk was higher than that in the spring milk, which means that the results regarding the cohesiveness of the curds do not confirm the conclusions of Chen et al. [67].

Springiness is defined as the ability of a sample to regain shape after the pressure force stops. According to the sensory definition, springiness is the distance from which a deformed sample will regain its dimension after the deforming forces cease [62]. The addition of 20 mg Ca (per 100 g of milk) in the form of citrate resulted in the increased springiness of the cheese in both seasons compared to the control sample. Cheese made from spring goat’s milk showed a better ability to regain shape after the pressure stopped compared to cheese from the spring-summer season. The ability to recover the shape of the spring season cheese from milk with 15 and 20 mg Ca 100 g^−1^ was significantly higher compared to the counterparts from the fall season (*p* ≤ 0.05) (Table 4). According to Rogers et al. [68], lower-fat cheese has a higher protein-to-fat ratio than full-fat cheese. Therefore, lower-fat cheese is characterized by a denser protein network and firmer texture. A more bonded protein structure may therefore be the reason for the higher springiness of lower-fat cheese. The results of our studies are different and indicate that cheese manufactured from autumn goat’s milk with a higher fat content had a lower springiness than cheese from the spring season. In a study by Chevanan et al. [69], adding calcium chloride to cheddar cheese increased its springiness.

Adhesiveness is a parameter characterizing the work required to overcome the forces of attraction between the surface of a food product and other bodies (tongue, teeth, or palate) with which it comes into contact during food consumption [70]. In most cases, the addition of calcium increased cheese adhesiveness compared to controls. The spring milk cheese with the addition of calcium citrate had significantly lower adhesiveness compared to its counterparts from the autumn season (*p* ≤ 0.05). A significant positive correlation coefficient was found between the adhesiveness of cheese with citrate in the autumn season and the calcium dosage (r = 0.6091; *p* ≤0.05) (Table 4). According to Zheng et al. [71], cheese adhesiveness is affected by temperature and fat content. When the temperature increases, the structure of full-fat cheese changes, the fat softens, and adhesion increases. Milk fat found as globules in the protein matrix network in cheese curd act as a plasticizer to inhibit the formation of cross-links between casein chains. These authors also reported that higher levels of potassium compounds increase adhesiveness, which might be correlated with the observed increase in fat globule size and pH values. An increase in adhesiveness was found in all goat cheese, with a concomitant increase in fat content. Cheese samples with the highest fat content also had the highest adhesiveness. Moreover, these cheese samples showed an increase in pH values with increasing dosage of calcium added to the milk. In contrast, Shabbir et al. [72] showed that adhesiveness decreased with lower moisture levels in goat cheese.

#### 2.3.4. Organoleptic Evaluation of Cheese

Many factors influence the sensory properties of cheese, including the hygiene of milk production and the selected processing technology [14]. Due to the specific organoleptic properties of goat’s milk and its goatish taste and odor, products made from it are not accepted by all consumers. According to Bohdziewicz and Jasińska [73], the fatty acids caproic, caprylic, and capric acids and short-chain butyric acid are responsible for creating a specific aroma in goat cheese. Some authors [74] claim that the off-odor and off-taste appearing in goat cheese resulted from the high-temperature processing of goat milk. The results of studies conducted by Park [21] and Znamirowska et al. [75,76] indicate that adding certain magnesium compounds could reduce the occurrence of unfavorable goat taste and odor in goat’s milk products.

The addition of calcium in the form of citrate did not significantly decrease the organoleptic characteristics of goat cheese. Similarly, calcium dose was not found to significantly influence the organoleptic characteristics of goat cheese. Additionally, the milk production season did not significantly affect the notes scored in the overall organoleptic evaluation of goat cheese (Table 5). The cheese was characterized by a slightly sour taste, fermentation and diacetyl odor, with a slightly noticeable goat smell. Chaudhari and Fanion [77] reported on the possibility of a chalky aftertaste in foods with calcium carbonate. Sheehan et al. [78] state that the odor and taste described as an animal (goat) or waxy is one of the most dominant characteristics of goat cheese. The goat acid cheese tested by Dmytrów et al. [38] obtained lower notes in the evaluation of taste, structure, and consistency compared to cow’s milk cheese. The authors stated that the notes for cheese made from goat’s milk given during the organoleptic evaluation were mainly due to an overly smeary consistency and a strongly perceptible goat-like odor. The slightly aromatic odor of fermented goat’s milk products is due to the lower content of volatile aromatic compounds (mainly diacetyl) and carbon dioxide formed during fermentation with mesophilic cultures. Szwocer et al. [79] consider goat’s milk to have a lower citrate content and thus a poorer composition of flavorings in fermented products. Therefore, adding calcium in the form of citrate could theoretically increase the diacetyl content of the cheese samples. However, the results of the organoleptic evaluation do not indicate the development of more aromatic compounds by the mesophilic bacteria used for fermentation.

## 3. Materials and Methods

### 3.1. Materials

Raw morning bulk goat’s milk for producing acid-curd cheese was collected in May (spring season) and September (autumn season) 2021 directly from an organic farm in southeastern Poland from different colored goats of mixed breeds.

The IBCm Bacto Kit 500 and IBCm SCC Kit reagents were purchased from Bentley Instruments Inc. (Chaska, MN, USA). Calcium citrate (Ca_3_(C_6_H_5_O_7_)_2_ · 4H_2_O) was purchased from Gadot Biochemical Industries Ltd. (Haifa, Israel).

The starter culture of mesophilic lactic fermentation bacteria G500 included strains of *Lactococcus lactis* subsp. *Lactis*, *Lactococcus lactis* subsp. *Cremoris*, *Lactococcus lactis* subsp. *Lactis*. Biovar *diacetylactis* was purchased from CSK Food Enrichment (Wageningen, The Netherlands). Beaugel 5 rennet was purchased from Coquard (Villefranche sur Saône, France) and the rennet strength was 1:3000 with 150 mg of active chymosin per liter.

All of the reagents used were of analytical reagent grade.

### 3.2. Goat’s Milk Analysis

Chilled raw milk (4 °C) before the analyses and cheese production was filtrated to remove dirt and foreign particles.

The total bacterial count (TBC) and somatic cell count (SCC) were determined in the BactoCount IBC M/SCC semi-automatic counter (Bentley Instruments Inc., Chaska, MN, USA). The chemical composition (protein, fat, lactose, and total solids) and freezing point were determined in milk and the milk product composition analyzer, Bentley B-150 (Bentley Instruments Inc., Chaska, MN, USA). The pH value was determined using a digital pH meter Toledo FiveEasy TM (Mettler Toledo, Greifensee, Switzerland) and an electrode InLab^®®^Solids Pro-ISM (Mettler Toledo, Greifensee, Switzerland).

The concentrations of macro- (Ca, K, Mg, P) and microelements (Mn, Se) in milk were determined using inductively coupled plasma—optical emission spectrometry (ICP-OES) method with a spectrophotometer Thermo iCAP Dual 6500 (Thermo Fisher Scientific Inc., Waltham, MA, USA) in mineralized milk samples according to the method of Znamirowska et al. [76]. The instrument was calibrated with standards (Merck, Darmstadt, Germany), including concentrations of 10000 ppm for macroelements and 1000 ppm for microelements. The Certified Reference Material (CRM) was used to validate analytical methods, and the recovery obtained for Ca was 98.00%, 99.00% for K, Mg—101.00% for Mg, 98.00% for P, 102.00% for Mn, 99.00% for Mo, and 102.00% for Se.

### 3.3. Control of the pH Value of Milk with Added Calcium after Pasteurization

Doses of calcium (5, 10, 15, 20, 25, 30, and 40 mg Ca 100 g^−1^ milk) added to raw goat’s milk in the form of citrate were tested. A total of 100 g of milk and the calcium dose calculated on the molecular weight of the calcium citrate were weighed. The samples were heated in a water bath to 25 °C. thoroughly mixed, and pasteurized at 90 °C for 15 s. A control sample without the addition of calcium was also prepared.

In the cooled samples of control milk (0 mg Ca 100 g^−1^ milk) with calcium addition (5, 10, 15, 20, 25, 30, and 40 mg Ca 100 g^−1^ milk), thermal stability was determined by observing the occurrence of protein denaturation and pH was measured using a FiveEasy pH meter (Mettler Toledo, Greifensee, Switzerland) with an electrode InLab^®®^Solids Pro-ISM (Mettler Toledo, Greifensee, Switzerland).

### 3.4. Acid Rennet Cheese Production

Each season (spring and autumn), the goat’s milk was divided into five batches (each 2000 g of milk), each with a different amount of calcium citrate added. Control milk (0 mg Ca 100 g^−1^ of milk) and milk with calcium addition (5, 10, 15, 20 mg Ca 100 g^−1^ of milk) were pasteurized (90 °C, 15 s). After the heat treatment, milk was cooled to 28 °C, and each group was inoculated with a 0.20% (*w*/*w*) starter culture of mesophilic lactic fermentation bacteria 15 min prior to the addition of 0.02% (*v*/*w*) of rennet. Then, the milk was thoroughly mixed and incubated at 28 °C until the pH reached 4.60 (±0.50). The obtained gels were cut into a cube (15 × 15 × 15 mm) and heated in a water bath at a temperature of 35 °C for 1.5 h. Next, the temperature was increased to 40 °C for 0.5 h. The whey was separated with cheesecloth and the curds were transferred to molds and pressed (10 N kg^−1^, 0.5 h) in a press with pneumatic cylinders (Pneumatig, Gdynia, Poland). Acid rennet cheese was cooled to 4 °C and stored in individual plastic containers for 24 h, after which analysis was carried out.

### 3.5. pH of Acid Rennet Curd and Cheese

The pH was determined in milk after pasteurization, in the acid rennet curds during coagulation, and in cheese with a pHmeter (FiveEasy Mettler Toledo, Greifensee, Switzerland) using an electrode InLab^®®^Solids Pro-ISM (Mettler Toledo, Greifensee, Switzerland).

### 3.6. Fat and Total Solids Content in Cheese

All physicochemical analyses were conducted on ground cheese samples to achieve uniformity. The fat content was measured according to the Gerber method [80]. The moisture content was calculated using the moisture analyzer MA 50.R (Radwag, Radom, Poland) according to the method of Kowalska et al. [81].

### 3.7. Degree of Protein Retention in Cheese

The content of protein in the milk and whey was used to calculate the degree of retention of protein in the cheese according to Siemianowski et al. [44].

The protein concentration in whey was determined in milk and milk products with the composition analyzer Bentley B-150 (Bentley Instruments Inc., Chaska, MN, USA).

### 3.8. Cheese Yield

Cheese yield was calculated based on the weight of cheese obtained relative to the weight of milk for cheese production and expressed in % [82].

### 3.9. Mineral Composition of Cheese

The mineral content of the cheese was measured as described in Section 3.2. ‘Goat’s Milk Analysis,’ with the following modification: 0.5 g of cheese was weighed.

### 3.10. Texture Analysis of Cheese

The TPA test determined the texture profile using a CT3 Texture Analyzer (Brookfield AMETEK, Middleboro, MA, USA) with the Texture Pro CT (Brookfield AMETEK, Middleboro, MA, USA) software. Cheese samples were cut into cubes (20 × 20 × 20 mm) and stored at 10 °C ± 1 °C before testing. Penetration testing was carried out using a plastic plunger type TA3/100 (25.4 mm diameter) with the following settings: distance 15 mm, measurement speed 5 mm s^−1^, and contact load 1.00 N [83].

### 3.11. Organoleptic Evaluation of Cheese

The organoleptic properties of cheese were evaluated according to the procedure of International Standards ISO4121:2003 [84]. Cheese samples were served in a random order in transparent plastic containers and coded with three-digit numbers. Twenty panelists (ten women and ten men, age 23–52) were selected to evaluate appearance and color, consistency, taste, odor and overall acceptability on a hedonic scale from 5 to 1 (5—extremely liked; 4—moderately liked; 3—neither liked nor disliked; 2—moderately disliked; 1—extremely disliked).

### 3.12. Statistical Analysis

From the obtained results, the mean, standard deviation and simple correlation coefficient (r) were determined statistically with the software Statistica 13.1 (StatSoft, Tulsa, OK, USA). The significance of differences between the averages was estimated with Tukey’s test (*p* ≤ 0.05). The experiment was repeated five times in each season. Twenty cheese samples were tested for each cheese variant.

## 4. Conclusions

This study confirmed that raw goat’s milk obtained in the spring season differs significantly in terms of cytological and microbiological quality and chemical composition, including micro- and macronutrients, from milk from the autumn season. Autumn milk was a richer source of total solids components, confirming the effect of the production season on milk quality. Moreover, the higher micro- and macroelements content of autumn goat’s milk resulted in significantly higher levels of minerals in cheese samples from the autumn season compared to the spring season.

The addition of calcium compounds is a widely used industrial practice in the manufacture of goat cheese, which can have a less firm curd due to goat milk’s properties. Calcium concentration is related to cheese texture, a crucial rheological property of cheese quality as consumers perceive it. An essential advantage of poorly water-soluble compounds such as calcium citrate is their neutrality to milk proteins, even at increased temperatures, so they can be added to milk before pasteurization without concern for decreasing the heat stability of proteins and precipitation. The use of calcium citrate at doses of 0–40 mg Ca in 100 g^−1^ milk did not reduce the thermal stability of goat milk proteins and allowed pasteurization to take place at 90 °C/15 s, indicating the potential use of this compound in goat milk processing. Moreover, this study showed the possibility of using calcium citrate to produce acid rennet cheese from goat’s milk.

The dairy industry is striving to reduce production losses, improve texture, and increase the yield of goat cheese. These benefits can be achieved through the better management of calcium in milk during cheese production. Calcium citrate is characterized by a high bioavailability of calcium and is therefore expected to increase in the production of cheeses with consumer-preferred health benefits.

## Figures and Tables

**Table 1:** Composition and physicochemical properties of raw goat’s milk.

Properties	Season	
	Spring	Autumn
TBC, cfu 1 mL^−1^	408,230 ^a^ ± 19,490	566,190 ^b^ ± 31,140
SCC, in 1 mL^−1^	1,032,220 ^a^ ± 33,370	1,608,490 ^b^ ± 45,910
Freezing point, °C	−0.571 ^a^ ± 0.017	−0,588 ^b^ ± 0.022
pH	6.67 ^a^ ± 0.03	6.62 ^a^ ± 0.07
Protein, g 100 g^−1^	2.69 ^a^ ± 0.29	3.26 ^b^ ± 0.23
Fat, g 100 g^−1^	2.83 ^a^ ± 0.48	3.52 ^b^ ± 0.27
Lactose, g 100 g^−1^	4.65 ^a^ ± 0.25	4.55 ^a^ ± 0.30
Total solids, g 100 g^−1^	10.29 ^a^ ± 0.42	11.63 ^b^ ± 0.70
Ca, mg 100 g^−1^	153.17 ^a^ ± 5.22	185.67 ^b^ ± 15.02
K, mg 100 g^−1^	178.57 ^a^ ± 1.49	207.84 ^b^ ± 2.00
Mg, mg 100 g^−1^	19.20 ^a^ ± 1.17	27.03 ^b^ ± 2.11
P, mg 100 g^−1^	128.11 ^a^ ± 3.34	144.87 ^b^ ± 2.56
Mn, µg 100 g^−1^	3.16 ^a^ ± 0.82	5.66 ^b^ ± 0.59
Mo, µg 100 g^−1^	5.20 ^a^ ± 1.12	7.80 ^b^ ± 1.01
Se, µg 100 g^−1^	3.14 ^a^ ± 0.78	4.75 ^b^ ± 0.45

Mean ± standard deviation; ^a,b^ means with different letters in rows indicate statistically significant differences at *p* < 0.05; TBC—total bacterial count; SCC—somatic cell count. Spring season: *n* = 25; autumn season: *n* = 22.

**Table 2:** Physicochemical properties and yield of acid rennet goat cheese.

Properties	Season	Calcium Dose, mg Ca 100 g^−1^ of Milk					r
		0	5	10	15	20	
pH	Spring	4.60 ^aA^ ± 0.01	4.61 ^aA^ ± 0.02	4.64 ^bA^ ± 0.01	4.64 ^bA^ ± 0.02	4.70 ^cA^ ± 0.05	0.7901 *
	Autumn	4.67 ^aB^ ± 0.04	4.67 ^aB^ ± 0.04	4.67 ^aB^ ± 0.03	4.70 ^aB^ ± 0.05	4.78^bB^ ± 0.03	0.6666 *
Fat, g 100 g^−1^	Spring	20.11 ^aA^ ± 1.67	19.42 ^aA^ ± 1.93	20.01 ^aA^ ± 1.64	19.50 ^aA^ ± 4.72	20.81 ^aA^ ± 4.06	0.2601
	Autumn	21.13 ^aA^ ± 1.55	20.82 ^aA^ ± 0.89	22.42 ^aA^ ± 0.78	22.15 ^aA^ ± 3.12	22.00 ^aA^ ± 0.85	0.4463
Total solids, g 100 g^−1^	Spring	30.82 ^aA^ ± 2.07	33.49 ^aA^ ± 1.67	34.00 ^aA^ ± 1.42	34.29 ^aA^ ± 2.13	34.49 ^aA^ ± 2.40	0.3857
	Autumn	36.16 ^aB^ ± 0.63	36.47 ^aA^ ± 2.92	37.81 ^aA^ ± 3.33	37.35 ^aA^ ± 3.63	40.18 ^aB^ ± 2.15	0.4299
Yield, %	Spring	15.23 ^aA^ ± 4.14	15.48 ^aA^ ± 3.09	15.80 ^aA^ ± 3.07	17.60 ^aA^ ± 3.13	17.60 ^aA^ ± 3.02	0.1263
	Autumn	22.08 ^aB^ ± 2.55	24.25 ^aB^ ± 4.98	23.58 ^aB^ ± 4.91	23.58 ^aB^ ± 2.00	23.60 ^aB^ ± 2.53	0.1233

Mean ± standard deviation; ^a–c^ means with different lowercase letters indicate statistically significant differences at *p* < 0.05 depending on calcium dose; ^A,B^ means with different capital letters indicate statistically significant differences at *p* < 0.05 depending on the season; r—correlation coefficient. *—statistically significant at *p* < 0.05; 20 samples × 5 batches × 2 seasons = 200 acid rennet goat’s milk cheese samples.

**Table 3:** Mineral composition of acid rennet goat cheese.

Properties	Season	Calcium Dose, mg Ca 100 g^−1^ of Milk					r
		0	5	10	15	20	
Ca, mg 100 g^−1^	Spring	120.83 ^aA^ ± 5.63	127.95 ^bA^ ± 2.80	130.15 ^cA^ ± 3.78	142.62 ^dA^ ± 5.64	147.45 ^eA^ ± 2.18	0.9823 *
	Autumn	130.66 ^aB^ ± 1.90	139.02 ^bB^ ± 3.30	142.01 ^bB^ ± 1.55	145.06 ^cB^ ± 3.90	151.21 ^dB^ ± 1.03	0.9367 *
K, mg 100 g^−1^	Spring	54.18 ^aA^ ± 10.49	56.26 ^aA^ ± 6.89	52.68 ^aA^ ± 4.32	52.61 ^aA^ ± 4.71	51.26 ^aA^ ± 5.21	–0.1222
	Autumn	55.73 ^aA^ ± 3.22	53.56 ^aA^ ± 2.26	55.05 ^aA^ ± 2.55	54.91 ^aA^ ± 3.01	54.15 ^aA^ ± 2.59	–0.3782
Mg, mg 100 g^−1^	Spring	14.72 ^aA^ ± 2.11	14.74 ^aA^ ± 1.09	14.53 ^aA^ ± 1.44	15.26 ^aA^ ± 2.54	14.70 ^aA^ ± 3.02	–0.0072
	Autumn	24.12 ^aB^ ± 2.72	21.37 ^aB^ ± 2.44	22.82 ^aB^ ± 2.85	22.71 ^aB^ ± 1.90	24.12 ^aB^ ± 3.14	–0.1257
P, mg 100 g^−1^	Spring	121.71 ^aA^ ± 1.02	125.88 ^abA^ ± 7.78	126.25 ^abA^ ± 7.90	133.87 ^bA^ ± 6.34	137.60 ^bA^ ± 7.12	0.7521 *
	Autumn	141.98 ^aB^ ± 3.63	141.39 ^aB^ ± 3.52	148.78 ^bB^ ± 2.78	151.96 ^bB^ ± 3.02	151.95 ^bB^ ± 3.32	0.7001 *
Mn, µg 100 g^−1^	Spring	6.23 ^aA^ ± 0.57	6.21 ^aA^ ± 0.11	6.14 ^aA^ ± 0.55	6.24 ^aA^ ± 0.14	6.25 ^aA^ ± 0.39	0.0112
	Autumn	6.61 ^aA^ ± 0.31	6.93 ^aB^ ± 0.35	7.02 ^aB^ ± 0.23	6.35 ^aA^ ± 0.10	6.99 ^aB^ ± 0.20	0.1145
Mo, µg 100 g^−1^	Spring	2.71 ^aA^ ± 0.39	2.71 ^aA^ ± 0.21	2.78 ^aA^ ± 0.12	2.81 ^aA^ ± 0.21	2.72 ^aA^ ± 0.17	0.1155
	Autumn	3.57 ^aB^ ± 0.10	3.58 ^aB^ ± 0.11	3.64 ^aB^ ± 0.12	3.61 ^aB^ ± 0.24	3.61 ^aB^ ± 0.21	0.2331
Se, µg 100 g^−1^	Spring	7.07 ^aA^ ± 0.56	7.55 ^aA^ ± 0.49	7.21 ^aA^ ± 0.48	6.01 ^aA^ ± 1.12	6.56 ^aA^ ± 0.44	0.0022
	Autumn	8.00 ^aB^ ± 0.67	8.25 ^aA^ ± 1.02	8.50 ^aA^ ± 0.90	8.22 ^aB^ ± 0.63	8.07 ^aB^ ± 0.85	0.1878

Mean ± standard deviation; ^a–e^ means with different lowercase letters indicate statistically significant differences at *p* < 0.05 depending on calcium dose; ^A,B^ means with different capital letters indicate statistically significant differences at *p* < 0.05 depending on the season; r—correlation coefficient. *—statistically significant at *p* < 0.05; 20 samples × 5 batches × 2 seasons = 200 acid rennet goat’s milk cheese samples.

**Table 4:** Texture of acid rennet goat cheese.

Properties	Season	Calcium Dose, mg Ca 100 g^−1^ of Milk					r
		0	5	10	15	20	
Hardness, N	Spring	2.16 ^aA^ ± 0.01	2.86 ^aA^ ± 0.57	2.89 ^aA^ ± 0.67	3.53 ^bA^ ± 1.42	3.45 ^bA^ ± 1.39	0.4662
	Autumn	2.26 ^aA^ ± 0.24	2.38 ^aA^ ± 0.30	2.88 ^aA^ ± 0.66	3.05 ^bA^ ± 0.69	3.16 ^bA^ ± 0.78	0.5481 *
Cohesiveness	Spring	0.30 ^aB^ ± 0.06	0.23 ^aA^ ± 0.14	0.26 ^aB^ ± 0.14	0.25 ^aA^ ± 0.06	0.33 ^aA^ ± 0.06	0.1675
	Autumn	0.18 ^aA^ ± 0.06	0.19 ^aA^ ± 0.11	0.19 ^aA^ ± 0.11	0.31 ^aB^ ± 0.01	0.27 ^aA^ ± 0.13	0.3754
Springiness, mm	Spring	3.56 ^aA^ ± 0.48	2.89 ^aA^ ± 0.71	3.26 ^aA^ ± 0.29	3.26 ^aB^ ± 0.28	4.40 ^aB^ ± 0.30	0.2959
	Autumn	3.06 ^aA^ ± 0.90	2.79 ^aA^ ± 1.48	2.87 ^aA^ ± 1.48	2.72 ^aA^ ± 0.52	3.22 ^aA^ ± 1.07	0.0353
Adhesiveness, mJ	Spring	1.52 ^aA^ ± 0.48	1.48 ^aA^ ± 0.33	1.25 ^aA^ ± 0.22	1.34 ^aA^ ± 0.32	1.59 ^aA^ ± 0.54	0.1293
	Autumn	1.76 ^aA^ ± 0.41	1.96 ^abB^ ± 0.27	2.23 ^abB^ ± 0.74	2.46 ^abB^ ± 0.27	2.55 ^bB^ ± 0.09	0.6091 *

Mean ± standard deviation; ^a,b^ means with different lowercase letters indicate statistically significant differences at *p* < 0.05 depending on calcium dose; ^A,B^ means with different capital letters indicate statistically significant differences at *p* < 0.05 depending on the season; r—correlation coefficient. *—statistically significant at *p* < 0.05; 20 samples × 5 batches × 2 seasons = 200 acid rennet goat’s milk cheese samples.

**Table 5:** Organoleptic evaluation of acid rennet goat cheese.

Properties	Season	Calcium Dose, mg Ca 100 g^−1^ of Milk					r
		0	5	10	15	20	
Overall acceptability	Spring	4.89 ^aA^ ± 0.19	4.84 ^aA^ ± 0.30	4.88 ^aA^ ± 0.16	4.77 ^aA^ ± 0.27	4.78 ^aA^ ± 0.52	–0.4689
	Autumn	4.83 ^aA^ ± 0.24	4.76 ^aA^ ± 0.23	4.75 ^aA^ ± 0.30	4.79 ^aA^ ± 0.27	4.79 ^aA^ ± 0.29	–0.0704
Appearance	Spring	5.00 ^aA^ ± 0.00	4.93 ^aA^ ± 0.26	4.97 ^aA^ ± 0.13	4.76 ^aA^ ± 0.37	4.60 ^aA^ ± 0.73	–0.3300
	Autumn	5.00 ^aA^ ± 0.00	4.75 ^aA^ ± 0.29	4.75 ^aA^ ± 0.29	4.88 ^aA^ ± 0.25	4.98 ^aA^ ± 0.25	–0.1581
Taste	Spring	4.92 ^aA^ ± 0.29	4.82 ^aA^ ± 0.41	4.78 ^aA^ ± 0.36	4.77 ^aA^ ± 0.46	4.50 ^aA^ ± 0.73	–0.2554
	Autumn	5.00 ^aA^ ± 0.00	4.75 ^aA^ ± 0.50	4.50 ^aA^ ± 0.71	4.63 ^aA^ ± 0.48	4.75 ^aA^ ± 0.29	–0.2044
Odor	Spring	4.96 ^aA^ ± 0.14	4.97 ^aA^ ± 0.13	4.99 ^aA^ ± 0.03	5.00 ^aA^ ± 0.00	4.99 ^aA^ ± 0.26	0.0130
	Autumn	5.00 ^aA^ ± 0.00	5.00 ^aA^ ± 0.00	4.75 ^aA^ ± 0.50	4.75 ^aA^ ± 0.29	4.88 ^aA^ ± 0.25	–0.2637
Consistency	Spring	4.79 ^aA^ ± 0.33	4.73 ^aA^ ± 0.56	4.87 ^aA^ ± 0.30	4.57 ^aA^ ± 0.59	4.47 ^aA^ ± 0.61	–0.2287
	Autumn	5.00 ^bA^ ± 0.00	4.63 ^aA^ ± 0.63	4.75 ^abA^ ± 0.29	4.75 ^abA^ ± 0.29	4.88 ^abA^ ± 0.25	–0.1232

Mean ± standard deviation; ^a,b^ means with different lowercase letters indicate statistically significant differences at *p* < 0.05 depending on calcium dose; ^A,B^ means with different capital letters indicate statistically significant differences at *p* < 0.05 depending on the season; r—correlation coefficient. *n* = 100.

## Data Availability

The original data presented in the study are included in the article, further inquiries can be directed to the corresponding author.

## Sample Availability

Samples of the compounds are not available from the authors.
